# Application of the rainbow trout derived intestinal cell line (RTgutGC) for ecotoxicological studies: molecular and cellular responses following exposure to copper

**DOI:** 10.1007/s10646-017-1838-8

**Published:** 2017-08-07

**Authors:** Laura M. Langan, Glenn M. Harper, Stewart F. Owen, Wendy M. Purcell, Simon K. Jackson, Awadhesh N. Jha

**Affiliations:** 1 School of Biological and Marine Sciences, University of Plymouth, Plymouth, PL4 8AA UK; 2 Electron Microscopy Unit, Faculty of Science and Engineering, University of Plymouth, Plymouth, PL4 8AA UK; 3 AstraZeneca, Alderly Park, Macclesfield, Cheshire SK10 4TF UK; 4 School of Biomedical and Health Care Sciences, University of Plymouth, Plymouth, PL4 8AA UK

**Keywords:** Ecotoxicology, Animal replacement, in vitro, Rainbow trout, RTgutGC, Dietary, Copper

## Abstract

There is an acknowledged need for in vitro fish intestinal model to help understand dietary exposure to chemicals in the aquatic environment. The presence and use of such models is however largely restrictive due to technical difficulties in the culturing of enterocytes in general and the availability of appropriate established cell lines in particular. In this study, the rainbow trout (*Oncorhynchus mykiss*) intestinal derived cell line (RTgutGC) was used as a surrogate for the “gut sac” method. To facilitate comparison, RTgutGC cells were grown as monolayers (double-seeded) on permeable Transwell supports leading to a two-compartment intestinal model consisting of polarised epithelium. This two-compartment model divides the system into an upper apical (lumen) and a lower basolateral (portal blood) compartment. In our studies, these cells stained weakly for mucosubstances, expressed the tight junction protein ZO-1 in addition to E-cadherin and revealed the presence of polarised epithelium in addition to microvilli protrusions. The cells also revealed a comparable transepithelial electrical resistance (TEER) to the in vivo situation. Importantly, the cell line tolerated apical saline (1:1 ratio) thus mimicking the intact organ to allow assessment of uptake of compounds across the intestine. Following an exposure over 72 h, our study demonstrated that the RTgutGC cell line under sub-lethal concentrations of copper sulphate (Cu) and modified saline solutions demonstrated uptake of the metal with saturation levels comparable to short term ex situ gut sac preparations. Gene expression analysis revealed no significant influence of pH or time on mRNA expression levels of key stress related genes (i.e. *CYP3A*, *GST*, *mtA*, *Pgp* and *SOD*) in the Transwell model. However, significant positive correlations were found between all genes investigated suggesting a co-operative relationship amongst the genes studied. When the outlined characteristics of the cell line are combined with the division of compartments, the RTgutGC double seeded model represents a potential animal replacement model for ecotoxicological studies. Overall, this model could be used to study the effects and predict aquatic gastrointestinal permeability of metals and other environmentally relevant contaminants in a cost effective and high throughput manner.

## Introduction

There is a great societal need for better understanding and monitoring of environmental contaminants discharged in the aquatic systems and their potential impact on the organisms (Donnachie et al. 2016; Jha 2008, 2004). Tissue and cell culture systems have been used for progressing the fundamental understanding of biological processes in controlled artificial environments, outside an animal’s systemic control (Heikkinen et al. 2010; Eisenbrand et al. 2002). Information obtained using these in vitro models aid in understanding ecotoxicological principles (Burden et al. 2015a; Castaño et al. 2003). With the development of these high throughput systems which could potentially replace the use of animal tests, the number of cell lines currently in existence is vast. The American Type Culture Collection (ATCC) currently holds over 4000 cell lines from 150 different species, with an unsurprising dominance of human derived cell lines (http://www.attc.org/). Fish cell lines have been useful in many areas of research, with their development originally to support aquatic animal viral diseases identification and treatment (Wolf and Quimby 1962).

Rachlin and Perlmutter (1968) first proposed the use of fish cell lines as an in vitro tool for the assessment of toxicity of environmental pollutants to aquatic biota, but it has only been recently that their use has grown tremendously. This is reflected in the wide variety of freshwater and marine species in addition to tissues of origin over an array of applications including fish immunology (Fierro-Castro et al. 2012), toxicology/ecotoxicology (Castaño et al. 2003), biotechnology and aquaculture (Kawano et al. 2011). In comparison to mammalian cell lines, fish cell lines are easier to maintain, manipulate, and produce highly reproducible results. In 1994, Fryer and Lannan reported some 159 fish cell lines (marine and freshwater), with Lakra et al. reporting a further 124 newly established cell lines by 2011. Despite the widespread need for replacement of animals in toxicity testing across all scientific disciplines, environmental contaminants such as metals, pharmaceuticals or chemicals are rarely assessed in vitro, not even for screening purposes. However, with the strict implementation of the 3Rs (*Reduce, Refine and Replace*) approach, societal and ethical constraints in addition to economic implications, there is a growing demand for in vitro assays or other procedures to reduce the number of fish used for assessing the ecotoxicity of chemicals. Recently, there has been a combined effort from academia and industry to propose new strategies to reduce the number of fish in acute toxicity tests (Burden et al. 2015a, 2014; Scholz et al. 2013; Hutchinson 2008; Jeram et al. 2005). Burden et al. (2015b) summarises several initiatives currently under way to improve confidence in newer alternative methods, which will support a move towards a future where less data from animal tests is required in the assessment of chemical safety. There is an opportunity to further this trend with the search for appropriate in vitro models of different organ systems to better understand the physiology of fish.

The intestine of a fish is a multifunctional organ (Jutfelt 2011; Grosell et al. 2010) responsible for the absorption of nutrients, ionic and osmotic regulation in addition to functioning as a barrier to keep unwanted agents such as a pathogens, toxins and microorganisms out. Knowledge of fish intestines has been obtained through a variety of different means using a large number of animals, with uptake/absorption studies primarily addressed using either flow through systems or through the use of short term *ex situ* methods such as the”gut sac” model. This well used technique allows the manipulation of both mucosal and serosal solutions and has been employed to understand mechanistic action of metal antagonists in fish (Nadella et al. 2011; Ojo and Wood 2007; Nadella et al. 2006a, b), in addition to pharmaceutical uptake in other organisms (Dixit et al. 2012; Mariappan and Singh 2004). However, it is potentially limited in terms of reduced cell viability, loss of enzymatic activity and limited exposure and sampling time (2–4 h) (Alam et al. 2012), in addition to requiring the sacrifice of an animal. In terms of xenobiotic metabolism, or its protective function against toxic action, knowledge of key factors governing xenobiotic/toxicant metabolism is far from complete.

Currently, intestinal epithelial models are based on the culture of a suitable cell type directly on flat, porous supports such as Transwell inserts. Among the available models, Caco-2 cell monolayers is one of the best studied approaches and is considered the gold standard for predicting in vitro intestinal permeability and absorption for mammalian studies (Vllasaliu et al. 2014; Gupta et al. 2013; Hubatsch et al. 2007; Gan and Thakker 1997; Bailey et al. 1996). Intestinal cells, such as the Caco-2 cell line, are typically grown single seeded on Transwell inserts and allowed to differentiate for up to 21 days prior to experiment initiation. However, the Caco-2 cell culture method has had numerous improvements proposed (Ferruzza et al. 2012; Galkin et al. 2008; Anna et al. 2003; Yamashita et al. 2002) to overcome the variability and heterogeneity visible in the literature in terms of performance (for review see Sambuy et al. 2005). Although little information is currently available in the literature, double seeding of the same cell line might reduce the requirement for extra nutrients or expensive additives allowing for the development of polarised, differentiated cells in a comparatively shorter time facilitating potential future high throughput requirements. Indeed, the use of double seeding techniques is a common practice in cell culture methods of fish epithelial cells (Schnell et al. 2016; Stott et al. 2015; Wood et al. 2002).

There is currently one available intestinal cell line derived from the rainbow trout, *Oncorhynchus mykiss* (Kawano et al. 2011), but our knowledge of this cell line is far from complete. Active transport mechanisms in the form of ATP binding cassette (ABC) transporters have been confirmed (Fischer et al. 2011) in addition to major-histocompatibility genes (Kawano et al. 2010). However, to our knowledge, its ability to function as an in vitro toxicity tool is limited to two studies. Catherine Tee et al. (2011) investigated the response of the RTgutGC cell line to a contaminant in the form of a dark blue colorant (Acid Blue 80) exposed to a monolayer, but found another cell line to be more sensitive while Geppert et al. (2016) investigated nanoparticle transport in the cell line using a two-compartment barrier model. While nanoparticle uptake was confirmed in this model, it is interesting to note that the standardised methodology of the Caco-2 cell line was employed, namely the growth of the cells over a 21 day period.

Metal metabolism within an organism has a significant effect on their accumulation, distribution and toxicity, with fish known to be particularly sensitive to many waterborne pollutants. Copper (Cu) is a ubiquitous major toxicant in the aquatic environment, and of greater environmental concern compared to other contaminants such as pharmaceuticals (Donnachie et al. 2016). It is also recognised as one of the best-studied metal micronutrient transport systems in the fish intestine (Bakke et al. 2010) with information primarily obtained from live animal in vivo feed trials and not in vitro experiments. As the relationship between Cu uptake in the intestine of rainbow trout is well established, we use this metal to probe the comparability of the cell line to the gold standard “gut sac” method already published (for example Nadella et al. 2006b).

In the culture of gill cells, a single seeding technique was initially employed (Parton et al. 1993), but was later adapted to a double seeding technique to improve attachment signals and surface structures (Fletcher et al. 2000). It is now employed as the standard culture method for gill cells (Schnell et al. 2016; Stott et al. 2015). Although a single seeding technique has previously been employed with the RTgutGC cell line (Minghetti et al. 2017, Geppert et al. 2016), we postulate that the application of a double seeding technique with this intestinal model would increase the complexity and therefore efficiency of the model making it more comparable to observations from “gut sac” experiments. A well-established critical step towards the use of in vitro assays as models for in vivo animal experiments is the correlation between in vitro and in vivo activities. In light of the outlined information, our objectives were to investigate the application of the double seeding technique to a fish intestinal cell line (RTgutGC). We hypothesized that an increase in seeding density and double layer of the cells would provide more physiologically relevant intestinal signals and surfaces. This would take the form of polarised microvilli, presence of mucosubstances, tight junction formation, transporters such as p-glycoprotein in addition to other metabolic enzymes. After the thorough characterisation of the model’s basic structure, the ability of the RTgutGC cell line to tolerate the application of saline in the apical compartment with minimum adverse effects was investigated. Following the establishment of the intestinal cultures, biological responses following environmentally level of exposures to Cu was determined in terms of cellular viability, cytotoxicity, genotoxic and gene expression responses in order to probe the robustness of the model.

## Materials and methods

### Experimental design

**Fig. 1:**
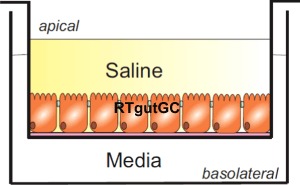
Outline of a typical single seeded Transwell culture system. The presence of two compartments (i.e. apical and basolateral) allow for the development of additional complexity into in vitro culture systems and exposure to environmental solutions. The system is widely used in human intestinal cultures and has been successfully employed using other cell types and double layers such as fish gills (Schnell et al. 2016, Stott et al. 2015). In the current study, the apical culture medium was substituted with a complex saline solution to investigate the compatibility of the cell line used (i.e. RTgutGC) for the assessment of environmental mixtures in future studies

Experiments were carried out in two stages, with stage I establishing the prerequisite requirements of an intestinal in vitro model (i.e. epithelial growth, mucosubstances, TEER (transepithelial electrical resistance) polarised micro-villi). This was followed by stage II in which the evaluation of Cu uptake by the in vitro intestinal model was performed in order to demonstrate comparability to the well-established “gut sac” method. Basic characterisation of the cell line was carried out to determine morphological characteristics, cellular viability, lactate dehydrogenase (LDH) level and genotoxic response (as determined by alkaline single cell electrophoresis or comet assay). Following basic characterisation as mentioned above, in stage II, Cu uptake by the model was determined using an analytical technique (i.e. ICP-OES) to demonstrate comparability to existing “gut sac” recordings (Nadella et al. 2006b). This was complemented by transcriptional expression analyses of the key genes involved in metal metabolism and stress response using RT-PCR. The selection of Cu concentration (i.e. 3 and 63 μM) represented non-toxic nominal concentrations typically found in the supernatant or lumen of intestinal compartment (Nadella 2006a, b). In order to make the in vitro model biologically relevant, cells were grown on Transwell inserts with cells seeded in layers (Fig. 1).

This Transwell system allows the growth of a monolayer in the apical compartment, with modifications of the media in both the apical and basolateral compartment possible (Fig. 1). The purpose of this study was to identify how this model compares with “gut sac” preparations. The double layering discussed in later sections was accomplished through seeding one layer initially, allowing cells to attach and grow, and then seeding again with more cells so that the first layer acts as scaffold thereby allowing cells to differentiate fully in a shorter period of time. Until now, this particular system has not been widely used in intestinal human cultures, but has been successfully employed using other cell types such as gills (Schnell et al. 2016, Stott et al. 2015). In addition, the model was tested for the ability to tolerate complex saline solutions by substituting medium in the apical compartment for this solution. This was investigated to establish whether the cell line would be capable of being used with environmental samples in the future.

### Chemicals and reagents

Leibovitz 15 medium (L-15), Dulbecco’s phosphate buffered saline (DPBS), trypsin, versene and Foetal bovine serum (FBS) were purchased in stock batches from ThermoFisher Scientific (UK). Plasticware in the form of T-75 cm^2^ flasks were exclusively obtained from Greiner Bio One (UK) while Transwell inserts were purchased from VWR (734-0051; Corning, UK). All chemicals were obtained from Sigma unless otherwise stated including Copper sulphate (CuSO_4_·5 H_2_O). Prior to the experiment, Cortland saline (Klinck and Wood, 2011) was prepared, pH adjusted to 7.7 and 7.4 (HCl), filter sterilised, aliquoted into 500 mL bottles and autoclaved for later use with cell culture. Osmolality of exposure solutions (L-15 and L-15:saline) was 274 mOsm and 204 mOsm, respectively, measured via a 5004 *µ* Osmette micro osmometer (Precision Systems, Massachusetts, USA).

### Cell culture

The rainbow trout gastrointestinal cell line RTgutGC (Kawano et al. 2011) was a kind gift from Dr. Lucy Lee (University of Fraser Valley, Canada). The cell line was routinely cultured in 75 cm^2^ culture flasks at a seeding density of 5 × 10^4^ cells/mL in an incubator set at 21 °C in L-15 culture medium supplemented with 10% FBS as per Kawano et al. (2011). All experiments were carried out in a controlled incubator set to 21 °C with non-parallel passages (17-37). A passage cut off of 37 was employed due to deviation from normal growth morphology. During this study, higher seeding densities than previously employed with the RTgutGC cell line were used. Both Minghetti et al. (2017) and Geppert et al. (2016) reported using 62,500 cells per cm^2^ under single seeding conditions (grown for 21 days) while our study employed 89–179,000 cells per cm^2^ (grown for 5 days) dependent on single or double seeding methodology. Kawano et al. (2011) previously observed that under super confluent conditions, RTgutGC cells stain intensely for alkaline phosphatase (an indicator of cellular differentiation) suggesting that high seeding densities may affect the structural and functional properties of the intestinal monolayer. This trend has previously been observed in the Caco-2 cell line (Natoli et al. 2011), where it is typically grown for 21 days to allow full differentiation.

Prior to Cu exposure experiments, cells were first grown both single seeded and double seeded on Transwell inserts to identify the variability between models over a short time period (9 days) (Fig. 1). Cells were seeded at a density of 1 × 10^5^ cells per mL of conditioned media for single seeding. With respect to double seeding, cells were again seeded at a density of 1 × 10^5^ cells/mL (∼89,285 cells per cm^2^) and allowed to attach for 24 h. After 24 h, 500 µL of the media in the apical compartment was replaced with secondary flask (same passage) at a seeding density of 2 × 10^5^ cells/mL, giving a final seeding density of 2 × 10^5^ cells (178,571 cells per cm^2^) in 1 mL of cell culture media. Cells were grown for 9 days initially to identify stabilisation of TEER measurements, with medium changes to both apical and basolateral media performed every 48 h.

### Morphological characterisation

RTgutGC cells grown on Transwell inserts for 5–7 days were fixed with 4% formol saline for 1 h, and stained with periodic acid and alcian blue to assess presence of mucosubstances. To stain for tight junctions, cells were grown as monolayers on coverslips and processed as normal as previous studies have shown no difference in immunofluoresence staining of cells on coverslips vs. Transwell systems (Gillespie et al. 2016). The tight-junction protein zonula occludens 1 (ZO-1) and E-cadherin (E-Cad) were detected using polyclonal antibodies goat anti-mouse (1579585; ThermoFisher) and goat anti-rabbit (1583138: ThermoFisher) as per Gendron et al. (2011). The secondary antibodies used were Alexa Fluor 594 (10644773; Fisher Scientific, UK) and Alexa Fluor 488 (10729174; Fisher Scientific, UK) at a concentration of 10 µg/mL. Cells were counter stained with DAPI to stain nuclei. Images were obtained using a Nikon epifluoresence microscope (Eclipse 80i) with camera attachment (DS-Qi1Mc). Images were captured and processed using the NIS elements application suite (Nikon). Finally, double seeded cells were allowed to grow until confluent (7–9 days) on a Transwell insert and fixed in 2.5% glutaraldehyde (in 0.1 M sodium cacodylate buffer; pH 7.2). Cells were washed with buffer (0.1 M sodium cacodylate; pH 7.2) and then secondary fixed with Osmium Tetroxide (1 h). After buffer washes, samples were dehydrated through grades of ethanol, and resin embedded (in Agar low viscosity resin). The resulting block was sectioned with a Leica Ultracut E ultramicrotome using a Diatome diamond knife (Agar Scientific; Stanstead UK), with sections (80 nm thick) transferred to a 200 µm mesh thin bar copper grids (Agar Scientific, UK). Sections were stained with uranyl acetate and Reynolds lead citrate and images captured on a JEOL 1400 TEM using a variety of magnifications. An accelerating voltage of 120 kV was used to capture images using a Gatan Orius camera.

### Transepithelial electrical resistance (TEER)

As single seeding of RTgutGC cells has previously been carried out (Geppert et al. 2016), we first established the comparability of single (SSI) and double seeding (DSI) using TEER and chopstick electrodes. Cells were seeded onto a permeable polyethylene (PET) membrane inserts with 0.4 µm pores with a surface growth area of 1.13 cm^2^ (Corning, UK) and maintained at 21 °C. The development of an intact intestinal epithelium was monitored daily through blank-corrected measurements of TEER using an EVOM epithelial voltohm-meter (World Precision Instruments, Hertfordshire, UK) fitted with chopstick electrodes (STX-2). As discussed in Results, TEER, with culture medium on both surfaces, increased minimally in both the SSI and DSI preparations, both reaching a stable plateau at approximately 6–9 days. Following the identification of a similar trend between the two models, the double seeded technique was hence employed in the following experiments, with exposures carried out from day 5 (Cu dosing).

As discussed in Results, Low TEER values suggested that the RTgutGC cell line may represent a “leaky gut” environment where resistance measurements may be very small. Indeed, the presence of variability between the methods outlined above would suggest the need for a more sensitive tool to measure TEER. To optimise the reliability of TEER measurements, dosing experiments used the more sensitive Endholm 12 culture cup, which is designed for epithelium with low TEER values. Experimental cultures were established as outlined below in section Experimental conditions. Appropriate blank corrections were determined for each experiment and for each TEER recording from inserts with no cells and incubated with appropriate apical (saline or L-15) and basolateral solutions identical to those used in experimental preparations (L-15 with minimal FBS). All experiments and exposures were based on 2-3 inserts derived from one biological replicate equivalent to one passage of the RTgutGC cells. Results are representative of three experiments with TEER values given as Ω cm^2^.

### Experimental conditions

Prior to experiment, cell viability was assessed using the trypan blue cell exclusion assay, with a viability of >98% deemed appropriate for future experimentation. Cells were seeded as outlined in previous sections and media exchanged after 48 h (full basolateral and half apical). A half media exchange (unconditioned L-15 medium) was carried out on day 4 of cell growth (apical and basolateral). In preliminary work carried out prior to the experiment, the cell line was found to be able to tolerate apical saline for a period of 24 h, but at a ratio of 1:1 (L-15:saline) is capable of tolerating it for a period of 96 h without adverse effects in terms of cell viability or LDH [data not included]. Due to this capacity, the experimental design allowed for probing the modification of the pH of the apical media to physiologically relevant ranges (i.e. pH 7.7 and 7.4). This pH range relates to the mid and posterior intestinal regions respectively (Fard et al. 2007) which could modify the cell line behaviour in terms of uptake and other biological responses to a more region specific response. Hence, on day of exposure 500 µL of medium was removed from all apical compartments, and exchanged for 500 µL of controls (unconditioned L-15:saline pH 7.7/7.4) or Cu consisting of the control solution spiked with 6 and 126 µM. Stock concentrations of Cu (in DPBS) were made prior to the experiment, with experimental concentrations measured prior to each experiment by inductively coupled plasma mass spectrometry (ICP-OES; iCAP 7000 Series ICP spectrometer, Thermo Scientific, USA) with Cu standards from ThermoFisher (UK). All biological responses (i.e. LDH measurements, genotoxic response, Cu uptake and gene expression) were carried out on Transwell inserts under double seeding conditions except for quantification of tight junction formation and cell viability, which were grown as single seeded monolayers. Characterisation of response of cells to Cu began with exposure on day 5, with the first sample recorded on day 6 (i.e. after 24 h or day 1 of exposure) and thereafter every 24 h for the duration of the experiment. To aid in comparisons to literature, the exposure conditions will henceforth be referred to in hours (24, 48, 72 h) to denote time elapsed post exposure and avoid confusion with period of time to culture.

### Analysis of biochemical and genotoxic responses

#### Determination of cell viability

Cell viability was assessed using the acid phosphatase assay (APH) as per the methodology of Friedrich et al. (2007, 2009). Cells were seeded in 96 well plates at similar seeding densities to the double seeded insert model (scaled for volume difference between plastic-ware) and allowed to grow for 5 days prior to exposure. This is equivalent to a seeding density of 40,000 cells per well of a 96 well plate (200 µL volume). Cells were exposed as outlined in Experimental conditions, washed with Dulbecco’s phosphate buffered saline (DPBS) prior to the addition of Acid phosphatase buffer (APH) containing 0.1 M sodium acetate, 0.1% Triton X-100 supplemented with *p*-nitrophenyl and incubated for 4 h at 21 °C in the dark. Following incubation, 10 µL of NaOH was added to each well to stop the reaction and absorbance measured on a spectrophotometer (FLUOstar Omega, BMG Labtech, UK) at 405 nm. Data was expressed as a percentage of control (unexposed cells in L-15 media) after correction for fluorescence from incubation buffer.

#### Determination of lactate dehydrogenase (LDH) activity

Concurrent to the collection of media from apical compartment of cells grown on Transwell inserts for analysis of Cu uptake, 200 µL of exposure medium was collected from surplus fluid at each sampling time and analysed for extracellular stress using the LDH assay as per Scholz and Segner (1999). Briefly, 50 µL of the media/saline aliquot was added to each well of a 96-well micro-plate in triplicate on ice and incubated with 250 µL of reaction buffer (50 mM TRIS/HCL, 0.14 mM NADH; pH 7.5) for 5 min at room temperature. Following incubation, the reaction started with the addition of 25 µL of 12.1 mM sodium pyruvate dissolved in 50 mM TRIS/HCL buffer (pH 7.5). Plates were briefly mixed and the enzyme activity recorded for 20 min at 25 °C in a micro-plate reader (FLUOstar Omega, BMG Labtech, UK) at 340 nm. Enzyme expression was subsequently standardised to cell counts.

#### Determination of genotoxic response

Genotoxic response following Cu exposure was assessed using single gel electrophoresis or comet assay and performed as per previously described for fish cell lines (Papis et al. 2011, Reeves et al. 2008, Nehls and Segner 2005, Raisuddin and Jha 2004). Prior to Cu exposure, the assay was validated using hydrogen peroxide as a reference genotoxic agent [data not included]. Briefly, cells were cultured as normal for 5 days and exposed to Cu as outlined previously.

Cells were removed from the wells using trypsin, with cell viability assessed using the Trypan Blue exclusion assay revealing an average viability of 97–98% (data not included). A subset of this cellular suspension was re-suspended in 1.5% normal melting point agarose, covered with a coverslip and dried at 4 °C. Slides were immersed in lysis solution for 1 h and then placed in electrophoresis tank to unwind (Compac-50 HTP Comet Assay Tank, Cleaver Scientific, UK). Electrophoresis was performed at 25 V, 620 mA for 25 min. Sliders were scored using an epifluoresence microscope (DMR; Leica Mi- crosystems, Milton Keynes, UK) and imaging system (Comet IV, Perceptive Imaging, UK) where 50 cells per microgel (100 cells per slide) were analysed per treatment. Slides were coded and randomised to ensure unbiased scoring. Comet assay software packages record a number of different parameters, with % tail DNA considered the most reliable (Kumaravel and Jha, 2006). Hence, comet assay results are reported as % tail DNA.

### Copper uptake using ICP-OES

For analysis of Cu uptake in the Transwell intestinal system, the experimental design consisted of three concentrations in the apical compartment of the Transwell system (L-15: saline control, 3 and 63 µM) over a 72 h period. Exposure concentrations chosen represent nominal concentrations of Cu found in the supernatant of gut contents and have been associated with reported standard fish farm diets between 5–70 µM L^*−*1^ (Nadella et al. 2006b), though these concentrations are dependent on the region of the gut sampled (Ojo and Wood et al. 2007). Medium was removed from the apical (1 mL) and basal (2 mL) compartment of the insert and analysed separately to account for active transport of the Cu between the apical and basal compartment (24–72 h). Duplicate samples were analysed with calibration, reagent blanks and reference material (Cu) to check quality assurance and quality control at the beginning, during and at the end of each ICP-OES run. For each model, biological variability was incorporated by repeating the experiment in non-parallel passages, so henceforth all results presented are indicative of an *n* of 4 (passages 24–37). The accumulation/loss of Cu was analysed among the treatment concentrations (0, 3 and 63 µM), time and between exposure solution conditions (pH 7.7/7.4).

#### Copper (Cu) uptake analysis

Cu uptake (nmol cm^*−*2 ^h^*−*1^) was calculated based on Klinck and Wood (2011) with some small modifications. The uptake rate has been modified to represent

$$Jin = cpm\, \times \,{(SA\, \times \,t\, \times \,GSA)^{ - 1}}$$

Where, *cpm* now represents final concentration in ppm, SA represents the initial measurements (initial exposure taken from stock reagents), t is the flux time (how long they were exposed; 24, 48 and 72 h) and GSA is the insert surface in cm^2^. Uptake rate was based on supernatant collection from the apical compartment during exposure experiment and standardised to surface area of Transwell cup where a larger Cu concentration at the end would indicate reduced Cu metabolism in the model. Data was tested for assumptions and analysed using analysis of variance (ANOVA) with exposure concentrations, time course and pH of exposure solution as the main factors.

### RNA extraction and reverse transcription

Due to 3 µM Cu representing environmental background levels in aquatic systems, this concentration was used as the negative control in this experimental part. Total RNA was isolated from pooled Transwell samples (3) of double seeded RTgutGC cells using RNAzol RT (R4533; Sigma, Germany). RNA quality was assessed using a Nanodrop ND1000 (ThermoFisher, UK), and RNA concentration quantified using a fluorescence kit (Quant-iT RiboGreen; Life Technologies, UK) according to manufacturer’s instructions. Twenty nanograms of samples with OD_260_:OD_280_ >1.9 and crisp bands were used for reverse transcription with NanoScript2 Reverse Transcription kit with Oligo-dT primer and random nonamer primers (RT-nanoScript2; PrimerDesign, UK). RT-PCR was performed on samples in triplicate (Step-One Plus RT-PCR system, Applied Biosystems) on a 384 plate with reactions containing 5 µL of Syber Green, forward and reverse primers (supplementary information), reference dye and nuclease free water to a final volume of 10 µL per well and 2 µL template cDNA. Initial denaturation was 94 °C for 2 min, followed by 40 cycles of 94 °C for 15 sec and 60 °C for 1 min, with a melt curve to verify PCR-product purity. RT-controls and appropriate no-template controls were also run using sterile nuclease free water.

### RT-qPCR

Primer concentration was optimised prior to experimentation to improve performance of RT-PCR as suggested by manufacturer (Table S1). Relative expression ratio (RER) of 5 genes selected for the study (i.e. *CYP3A, mtA, SOD, GST* and *Pgp*) was calculated relative to a pseudo reference gene composed of *β -actin*, *ef1α* (elongation factor 1α) and *18 s* as per recommendations by Vandesompele et al. (2002) to mitigate relatively large errors while using a single reference gene. Amplification efficiencies of individual reactions were incorporated as per recommendations by Liu and Hu (2002), with PCR efficiency measured using LinRegPCR (Ramakers et al. 2003) relative to a”pseudo-housekeeper”. Data was analysed using the efficiency corrected method of Pfaffl (2001), with individual sample efficiency (calculated using LinReg) as has been applied in other RT-PCR analysis methods (Rao et al. 2013). Statistical analysis of gene expression data was carried out using the non-parametric Wilcoxon Rank Sum test on *∆* Ct value (Ct_*target*_ – Ct_*reference*_) as recommended by Yuan et al. (2006). This test was chosen due to its robustness with small sample sizes and lack of presumption regarding data distribution.

### Statistical analysis

Statistical analyses were performed in R Version 3.1.3 (RStudio T 2015). Data is given as the mean value ± standard error of the mean (SEM), with “n” denoting replicates (passages) per experiment. These replicates are representative of non-parallel passages of the RTgutGC cell line, with each recording representing of 2–4 technical replicates. All data was first tested for normality using the Anderson-Darling Normality test (AD) in addition to examination of QQ-plots, while homogeneity of variance was conducted using Levene’s test, and an appropriate parametric or non-parametric test was then applied. Data which did not meet the assumptions of normality for parametric tests were analysed using the Kruskal-Wallis test followed by Dunn’s pairwise posthoc test with Bonferroni correction. In addition to analysis of Relative expression ratio, correlations between gene’s were determined using Pearson’s correlation co-efficient. Due to the multiple factors, data was analysed using a 2-way ANOVA with Tukey’s pairwise comparisons as post hoc if test assumptions were met. As per Dallas et al. (2013), median values of % tail DNA were used for statistical analysis. For all statistical analyses, a value of *p* < 0.05 was considered significant.

## Results

### Morphological characterisation

**Fig. 2:**
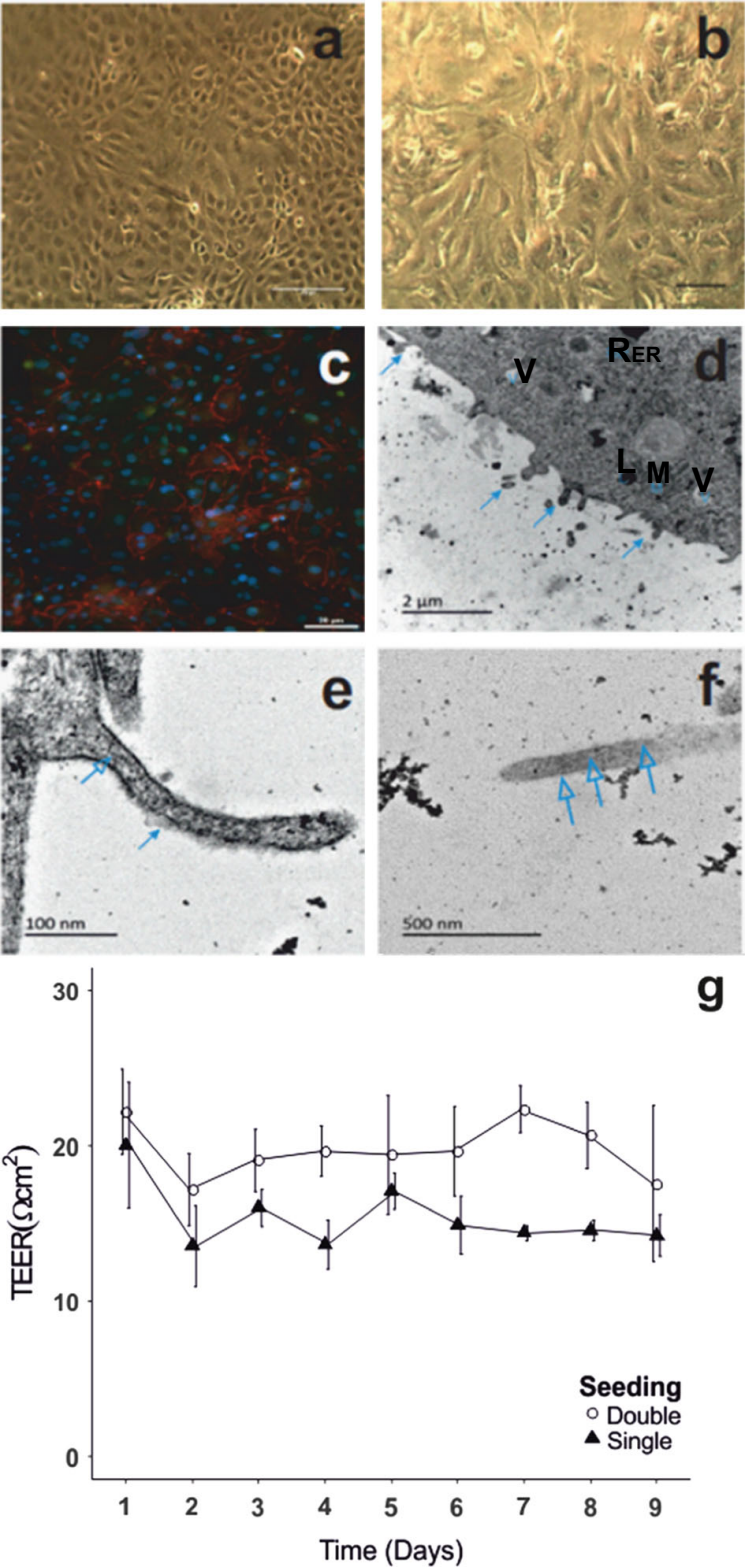
Characterisation of the double seeded intestinal fish cell model under in vitro conditions. **a** Characteristic epithelial growth of the RTgutGC cells after 7 days in single layer culture. **b** Double seeded RTgutGC cells grown on Transwell inserts showing weak positive staining for neutral mucosubstances indicative of active mucous secretion. **c** Immunofluoresence staining for ZO-1 (*red*) and E-cadherin (*green*) of double seeded RTgtuGC cells grown on Transwell inserts. Nuclei were counter stained with DAPI (blue). As expected, ZO-1 is expressed predominantly on the periphery of cells, while E-caderin is localised to the cell surface. **d–f:** Sub-cellular characterisation of double seeded RTgutGC cells confirms the presence of **d** polarised cells with microvilli protrusions [closed arrow heads]; Abbreviations: *Rer*   rough endoplasmic reticulum, *M*   mitochondria, *L*   lysosomes, *V*  vesicles and **e** fibrillary coats surrounding the protrusions [closed arrow head]; **f** Further confirmation of microvilli through the presence of a filamentous cytoskeleton running the length of the structure [open arrow head]. **g** Transepithelial electrical resistance (TEER) of RTgutGC cells seeded under double and single seeding conditions with no significant difference observed over time (*p* > 0.05) as measured using chopstick electrodes

The RTgutGC cells demonstrated typical epithelial morphology when grown as a single monolayer (Fig. 2a). Histological staining of the double-seeded monolayer revealed weak staining of neutral mucosubstances indicative of mucus secretion by the epithelial cells (Fig. 2b). Additionally, cells consistently (in both single and double seeded form) expressed the tight junction protein Z0-1 and E-cadherin supporting the identification of these cells as epithelial in nature (Fig. 2c). Examination of the ultrastructure of the double seeded cells revealed a polarised monolayer of cells grown for 5–7 days on Transwell supports (Fig. 2d). The untreated cells exhibit basally located nuclei and apical microvilli (Fig. 2d). These layers are rich in mitochondria, rough endoplasmic reticulum and tight junctions. The microvilli protrusions on the apical side of the membrane have been verified through the identification of a fibrillary coat or glycocalyx on the outside of the structures identified as microvilli and which were present even under saline exposure conditions (Fig. 2e). In addition, as support of these structures as microvilli, the filamentous cytoskeleton of the microvilli was observed extending into the monolayer cytoplasm (Fig. 2f). Cells developed a transepithelial electrical resistance (TEER) of 14 ± 1.33 Ω cm^2^ and 17 ± 5.02 for single and double seeded cells over 9 days respectively, with no significant difference observed in either model over time (*n* = 4, *p* > 0.05)(Fig. 2g).

### TEER

**Fig. 3:**
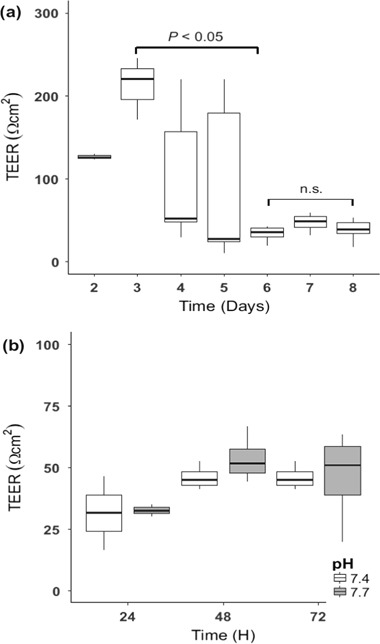
**a** Transepithelial electrical resistance (TEER) of the double seeded RTgutGC cell line over a 8 day period under symmetrical conditions (L-15 medium and 10% FBS in both apical and basolateral compartments) measured using the Endholm 12 culture cup system. Significant differences were only observed between day 3 and day 6, but not during the experimental time period (day 6–8). **b** Asymmetrical conditions where saline was applied to the apical compartment at a 1:1 ratio (L-15 medium: saline) and L-15 medium (containing FBS) was maintained as normal in the basolateral compartment. The application of saline to the apical compartment reveals a comparable trend in increased resistance (TEER) over time with a stabilisation after 72 h. This trend of stabilisation of TEER was also observed in L-15 medium (**a**) however no significant difference was observed between saline exposures (**b**). Significance was set at *p > *0.05

The replacement of the chopstick electrodes with the use of the static Endholm chamber resulted in an increase in baseline TEER measurements (Fig. 3a). However, the previously observed trend of a plateau in TEER following 6–9 days (Fig. 2g) was also observed using this method. As cells integrated following the double seeding event (initial seeding denoted as day 0, secondary seeding day 1), TEER resistance increased by approximately *∼*40% from 126.24 ± 2.96 Ω cm^2^ to 212.7 ± 37.6 Ω cm^2^ (*n* = 9) (Fig. 3a). Analysis of the data revealed non-normal data, with unequal variance. Significant differences were observed over time (*p* < 0.05, Kruskal-Wallis) with Dunn’s posthoc test revealing significant differences between day 3 and day 6 only (*p* < 0.05, Dunn’s test) (Fig. 3a). A direct comparison of TEER between medium (pH 7.5) and medium:saline (pH 7.7/pH 7.4) revealed an increase from 21.16 ± 0.68 Ω cm^2^ in L-15 alone to an average of 26.19 ± 4.77 Ω cm^2^ in saline on day 5 of sampling. This trend in increased TEER in medium:saline solutions vs. medium on its own is repeated when Cu exposures are also incorporated. No significant difference in TEER was observed during the exposure time of 6–9 days in L-15 alone (Fig. 3a), in the saline:medium combination (Fig. 3b), or in any combination of pH, concentration or sampling time.

### Analysis of biochemical and genotoxic response

#### Cell viability

**Fig. 4:**
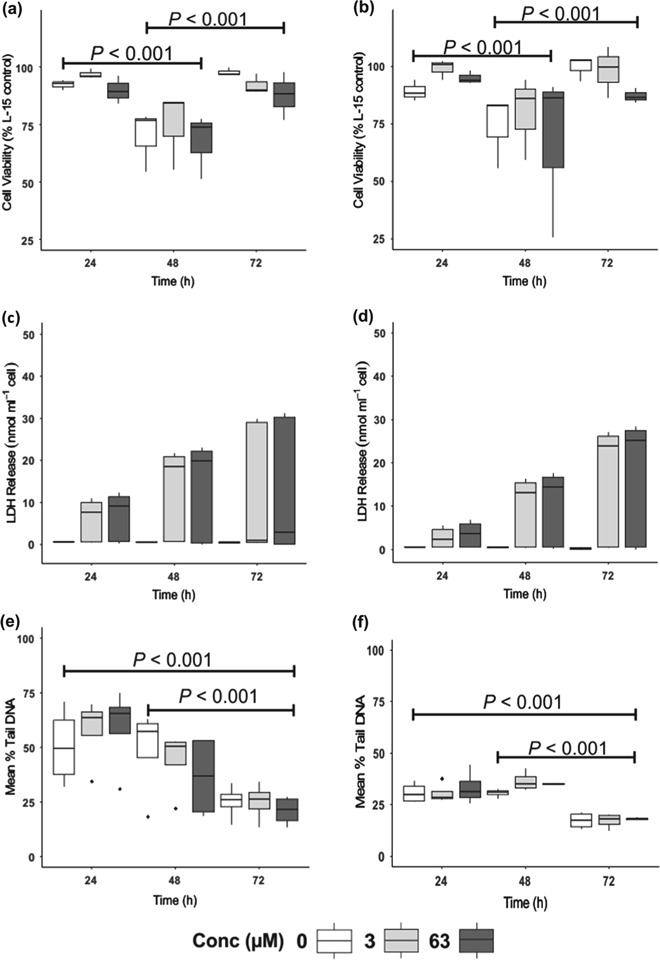
Comparison of changes in cell viability (APH) (**a** & **b**), LDH (extra-cellular) (**c** & **d**) and induced genotoxic damage (**e** & **f**) following a combined saline/Cu exposure. The legends located at the bottom of the graph denotes the Cu concentration levels, with the first graph of every assay representing pH 7.7 (equivalent to mid intestine) and the second corresponding graph representing pH 7.4 (equivalent to posterior intestine). Values are presented as the mean ± SEM, *n* = 3 – 4 biological replicates (passages) with 4 technical replicates per result. Significance was set at *p* *<* 0.05

In addition to preliminary screening of cellular viability using the Trypan Blue exclusion assay, three experiments were performed to analyze for variation in cellular viability using the APH assay following the multi-factor Cu exposure which took into account the confounding factors (e.g. pH, Cu concentrations and exposure time). The results of the cell viability assay were presented as percentage of the control following media autofluorescence blank correction (L-15 media with no exposure) and were logit transformed. Application of Anderson Darling normality test revealed normal data (*n* = 3, *p* > 0.05), with homogeneous distribution (QQ-plot). A two-way ANOVA revealed no significant differences between concentrations (*p* = 0.27) or pH (*p* = 0.92). Nonetheless, significant differences were observed between time which was consistent between assays (*p* < 0.001), with Tukey’s posthoc test identifying differences between 24 and 48 h cell viability (*p* < 0.001) and 48 and 72 h exposure (*p* < 0.001) (Fig. 4a, b).

#### Lactate dehydrogenase (LDH) activity

Four experiments were performed to determine damage to the cellular membrane following Cu exposure. Data was analysed using a Kruskal Wallis test due to non-normal data with unequal variances and found no significant differences between any of the factors (time, Cu concentration or pH). The data is presented was Fig. 4 c, d as the release of LDH into the media corrected by cell count.

#### Genotoxic response

Three experiments were performed to determine genotoxic response to Cu using our multi factor experimental design. Mean % tail DNA was used in analysis due to its normal distribution (*p* = 0.20 AD test). As noted in previous assays, no genotoxic response was found following Cu exposure. However, significant differences were identified over time (*p* < 0.001) with Tukey’s post hoc test identifying differences in response between 24 and 72 h (*p* < 0.001) and 48 and 72 h (*p* < 0.001) respectively (Fig. 4e, f), a trend also observed in the cell viability assay.

#### Copper uptake

**Fig. 5:**
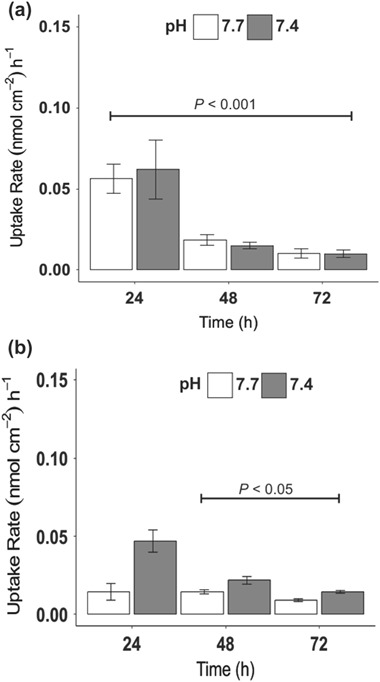
Copper (Cu) uptake rates in RTgutGC cell line double seeded on Transwell inserts. Robustness of the model was assessed through the application of Cu in the apical compartment at a 1:1 ratio of L-15 medium to saline at pH 7.7 (*white*) and 7.4 (*grey*). Data presented as mean ± SD over a 72 h sampling period. Statistical significance was tested using the Kruskal-Wallis test with Dunn’s test ran as posthoc. Significant differences found in Cu uptake between 24 and 72 h at 3 µM (**a**) and between 48 and 72 h following 63 µM (**b**) exposure. However, no significant difference was found between the two pH levels

Four experiments (*n* = 4) were performed to determine difference in Cu uptake rate dependent on pH of exposure solution in apical compartment of Transwell system. All samples used for Cu uptake had a baseline TEER value of >20 Ω cm^2^. Background concentrations of Cu in the Cortland saline solution at both pH 7.7 and pH 7.4 were 0.0468 ± 0.0022 ppm (0.73 ± 0.03 µM) and 0.01198 ± 0.00647 ppm (0.19 ± 0.10 µM) Cu respectively prior to exposure. Analysis of the data revealed non-normal data, with unequal variance. Significant differences were observed over time for both 3 µM (p < 0.001, Kruskal-Wallis) and 63 µM (p < 0.001, Kruskal-Wallis). Dunn’s posthoc test revealed significant differences between 24 and 72 h at 3 µM Cu exposure (p < 0.001, Kruskal-Wallis) (Fig. 5a), while significant differences were also observed between 48 and 72 h (p < 0.05, Kruskal-Wallis) for the 63 µM Cu exposure (Fig. 5b). A clear trend in response was visible at both concentrations, with the lower Cu concentration demonstrating a clear pH paired response. However, at the higher Cu concentration, the higher pH (7.7), representative of mid intestinal pH, clearly reached a plateau of uptake with limited difference in response over time (Fig. 5a). In contrast, the lower pH (7.4), representative of posterior intestinal pH demonstrated a comparable trend to observations at the lower Cu concentration (Fig. 5b). This trend in Cu-dependent uptake reiterates the functional properties of this model and reflects previous “gut sac” observations.

### RT-qPCR

The efficiency of RT-qPCR primers varied with individual samples. This was accounted for during analysis but typically ranged from 80–90% as assessed by the LinReg programme. The expression of five genes related to xenobiotic defence, metal and oxidative stress were investigated in the Transwell model under two apical pH exposure scenarios. Analysis of data revealed no significant difference between pH or as a function of time (*p* > 0.05). Pearson’s correlation analysis of gene expression on *∆* Ct values showed significant positive relationships between analysed genes as presented in Table 1.

**Table 1:** Pearson’s correlation coefficient of the expression of five stress related genes investigated in the study following exposure to Cu in the RTgutGC cell line

	CYP3A	GST	mtA	Pgp	SOD
*CYP3A*	1	0.91*	0.97*	0.95*	0.95*
*GST*	–	1	0.87*	0.88*	0.84*
*mtA*	–	–	1	0.98*	0.97*
*Pgp*	–	–	–	1	0.95*
*SOD*		–	–	–	1

Strong positive correlations were found in all combinations of genes. Astrix (*) denotes a *p* < 0.05

## Discussion

Whilst studies have been carried out to determine the toxicity of metals to intestinal tissue ex situ using”gut sac” methodology (Nadella et al. 2006b, 2007, 2011; Leonard et al. 2009; Ojo and Wood et al. 2007), there is little information on the response of intestinal cells under in vitro conditions. In the present study, we have established an improvement in the response of the RTgutGC cell line under double seeding conditions which is likely to better mimic the native physiology of the rainbow trout intestine than single seeding. RTgutGC cells expressed the weak presence of neutral mucosubstances characteristic of the distal region of the rainbow trout intestine, while intracellular tight junction formation was evidenced by pronounced staining for ZO-1 and E-caderhin, junctional complexes seen in the electron micrographs (TEM), and comparable TEER to in vivo intestinal reports (50–400 Ω cm^2^) (Jutfelt et al. 2006; Trischitta et al. 1999). Cellular morphology also appeared similar to differentiated enterocytes, which was confirmed as microvilli protrusions through the presence of a filamentous cytoskeleton connecting micro-villi to the underlying monolayer in addition to a fibrillary coat surrounding the protrusions. The retention of functional properties of tissue in established cell lines is not unique (Bailey et al. 1996, Lee et al. 2009). Unsurprisingly, this is reported more often in ex vivo primary cultures which are thought to retain more morphological and metabolic comparability to native fish tissues (Stott et al. 2015; Baron et al. 2012; Dowling and Mothersill, 2001). Based on morphological characterisation, the RTgutGC intestinal model appears to be well suited to study uptake and metabolism of metal and other contaminants in a dietary context.

In toxicological investigations using Transwell models, transepithelial resistance is routinely used as an endpoint for both cultured mammalian (Vllasaliu et al. 2014; Leonard et al. 2010; Sambuy et al. 2005) and fish gill epithelia cells (Schnell et al. 2016; Stott et al. 2015; Jonsson et al. 2006; Wood et al. 2002) and more broadly in permeability studies (Buckley et al. 2012). In this study, the TEER profile of the RTgutGC cell line demonstrated comparable resistant and trends to other intestinal derived cell lines with increased resistance following medium change and comparable baseline resistance to in vivo reports (50–400 Ω cm^2^) (Jutfelt et al. 2006; Trischitta et al. 1999). A previous study using the RTgutGC cell line in a Transwell system also reported TEER of approximately 33 ± 3 Ω cm^2^ on a 6 well Transwell insert after 21 days (Geppert et al. 2016), while we found a comparable TEER after only 5 days using the double seeding technique (average of 26 ± 5 Ω cm^2^ for both L-15:saline solutions). Direct comparison of both studies is difficult, as the larger the membrane or growth area of the culture cup, the lower the TEER measurements. In our study, TEER of RTgutGC cultures demonstrated a moderate increase over time as previously noted by Geppert et al. (2016). Interestingly, this trend in increased TEER was also repeated over time when L-15 medium was exchanged for the experimental solution of L-15:saline, highlighting its tolerance of the application of saline.

Examination of the literature on gill epithelia has attributed increased TEER following replacement of media with water/saline to a closure of ion channels in response to reduced sodium chloride in the apical membrane (Jonsson et al. 2006; Fletcher et al. 2000; Wood and Part 1997). However, in the current study there were minimal changes in sodium concentration between the two exposure solutions and no significant differences between controls and exposures (i.e. no difference between L-15 and L-15:saline). In the context of the intestinal system, sodium is a known osmotic regulator and when combined with a hypertonic solution can have a major impact on cellular resistance and permeability. Indeed, the reason that TEER may have increased in the study model when exposed to saline may be due to the decreased osmolality of the exposure solution (from 274 mOsm in L-15 medium to 204 mOsm in combination solution) and the minimal decrease in sodium levels (approximately 69 nM to 44 nM). Previous studies support this hypothesis in human intestinal models (Inokuchi et al. 2009; Noach et al. 1994). Using a hypotonic solution of 200 mOsm, Noach et al. (1994) observed that after application to HT29-cl19A cells, no significant change in TEER were observed following treatment apically. Instead, a clear increase in resistance was observed (*∼*144%), something which the fish intestine and human colorectal adenocarcinoma cell line have in common, although the degree of increase is substantially different between pH 7.7 (*∼*168%) and pH 7.4 (*∼*58%).

The present study hypothesised that the range of Cu concentrations used would not induce significant changes in biochemical responses or TEER responses. Previous studies of the uptake of Cu via the intestine have shown regions of this organ to become supersaturated above a threshold of *∼*63 and 157 µM for the mid and posterior intestine as demonstrated using the “gut sac” model (Nadella et al. 2006b). It has been postulated that the presence of this threshold (which demonstrates a maximum quantity which the cells can efficiently absorb) will cause a reduction in the toxic action of this metal on the apical/lumen membrane of this organ. Indeed, one drawback with the gut sac approach is the potential for hypoxia in this ex situ model, an area of great interest in vitro with 3D organoid models such as spheroids, where it has been difficult to measure the oxygen in the larger tissue structures (Langan et al. 2016). Using a variety of biochemical parameters (i.e. LDH, cell viability and DNA damage) and later analysis of Cu uptake, the presence of this supersaturation threshold in the RTgutGC in vitro intestinal model is supported. Generally, LDH is used to detect membrane damage by toxic agents (Acikgöz et al. 2013; Jurišić and Bumbaširević 2008), with LDH activity expected to increase with prolonged toxic exposure through the displacement of calcium ligands and disruption of the membrane permeability as previously demonstrated in fish (Mazon et al. 2004; Bury et al. 1998). Comparable to other toxicological models which support the presence of this supersaturation threshold (Teodorescu et al. 2012, 2008; Antognelli et al. 2006), the current study demonstrates the existence of this threshold through a reduction in both LDH release and lack of significant difference in cell viability (APH assay) suggesting direct comparability to the in vivo tissue. Indeed, this comparability is further enhanced when DNA damage is incorporated into the characterisation. Higher concentrations of Cu are known to induce DNA damage in teleost species, either through dietary uptake or exposure via media/water (Mustafa et al. 2012; Sandrini et al. 2009). Unlike other studies which aim to induce a genotoxic response with very high concentrations of toxicants, our study was limited to environmentally relevant concentrations (Bakke et al. 2010). It is therefore not surprising that no significant induction of DNA damage was observed using the alkaline comet assay. This is in line with original expectations and provides an appropriate baseline for future investigations (Jha 2004). While no significant differences were observed based on Cu concentrations for any of the biochemical or DNA damage assays used during this study, a clear trend was apparent whereby significant differences were consistently induced as a function of time for both cell viability and genotoxic assays. The observable trend would suggest that these two parameters are inherently correlated and although not investigated in the current study, similar observations have been reported in other animal models and humans using a range of parameters (Dallas et al. 2013; Jha 2008).

In fish, Cu may be taken up from the diet via the intestine or aqueously (via the gill) and transported to the liver with differing Cu routes of exposure resulting in differential up-take and transcriptional responses (Mustafa et al. 2012; Minghetti et al. 2008). In long-term exposures, teleost intestine appears to be the second most important organ after liver to accumulate Cu when exposed through a dietary route (Mustafa et al. 2012). Fundamental understanding of Cu and other metal accumulation in this organ is therefore an important aspect in ecotoxicological investigations where in vitro models can play an important role. In this context, previous studies using the”gut sac” model have suggested that Cu uptake differs in different parts or regions of the rainbow trout intestine (Nadella et al. 2006a, b). However, Nadella et al. (2006b) observed no significant differences between mid and posterior intestine after a 2 h exposure. In our study, Cu uptake (3 µM) in the cell line covers a comparable range for the mid (pH 7.7; 0.056 ± 0.018 nmol cm^*−*2^ h^*−*1^) and posterior (pH 7.4; 0.062 ± 0.036 nmol cm^*−*2 ^h^*−*1^) in vitro intestinal model to that reported by Nadella et al. (2006b). In their studies, the typical rate was found to be 0.025 and 0.036 nmol cm^*−*2^ h^*−*1^ for mid and posterior tissue respectively. Interestingly, uptake rate is only significant over the sampling period which may denote the time necessary for intrinsic homeostatic mechanisms to bring uptake and export rates into equilibrium, as has previously been observed for both fish gill and intestine (Kamunde et al. 2002a). The considerable (but not significant) decline in Cu (63 µM) in the proposed in vitro model at pH 7.4 suggest that the posterior intestine is the most active site of Cu absorption using this animal replacement system. This suggestion of site of uptake is supported by in vivo observations made by other authors who have identified the posterior intestine as the most active site for unidirectional Cu uptake in juvenile rainbow trout (Kamunde et al. 2002a, Clearwater et al. 2000, Nadella et al. 2006a).

In general, the mechanisms of gastrointestinal interactions of metals in animals and fish are not clearly understood. It is known that the maintenance of Cu balance involves the strict regulation of uptake, distribution, detoxification and excretion in fish (Kamunde et al. 2002b). As such, our study investigated five key genes related to xenobiotic defence, metal and oxidative stress. In contrast to our preliminary hypothesis, no significant difference was found between the two pH conditions or as a function of sampling period. This we believe may be related to the levels of Cu used in the study and is further supported by observations of Kamunde et al. (2002b) who suggested that intestinal uptake of Cu may require a threshold for optimal performance, which are less effective when Cu levels are low. Although no significant differences were observed during our study, it is important to note the presence of metabolising enzymes suggestive of both Phase I (*CYP3A*) and phase II (*GST*) biotransformation capacity in this Transwell system. The prevalence of correlations for transcriptional expression of selected genes within our study implies a harmonious metabolic system in this intestinal model capable of first pass metabolism and protection. Previously, van Herwaarden et al. (2009), El-Kattan and Varm (2012) noted that interplay between *Pgp* and *CYP3A*, through the sharing of similar substrates and modulators (Hunter and Hirst, 1997), enabled highly efficient metabolism in humans and thus could have a profound effect on first pass elimination of drugs. This trend is also seen in other combinations of genes such as in metallothionein (*mtA*) and superoxide dismutase (*SOD*) which were also positively correlated in our study (r^2^ = 0.97). In agreement with the literature, we propose that these two genes play a key role in protecting and maintaining cellular functionality against metal induced toxicity, with Fang et al. (2010) proposing their function in maintaining cellular metabolic homeostasis. Knowledge of transcriptional expression is a logical addition to a more integrative comparison of in vivo and in vitro studies, and will allow the correct placement and choice of such a model prior to toxicity testing. While other environmentally relevant contaminants (e.g. pharmaceuticals) were not investigated in the current study, the expression of the aforementioned genes opens this model to further testing of other contaminants of concern.

A variety of experimental models have been developed to target toxicology in the aquatic environment and these are readily available to the scientific community. The complexity of these models spans the range of system intricacy from ecosystems to populations, whole animals with different developmental stages, in situ perfusions, ex situ organs, tissue slices, 3D-organoids, co-cultures, primary cultures and mono-cultures of immortalised cells lines such as the RTgutGC. It is axiomatic that each model has both advantages and disadvantages dependent on the scientific need. In order to summarise the current study, we first address where the RTgutGC model falls with other in vitro animal alternative models. Unlike the most commonly cited human in vitro model (i.e. Caco-2 cell line), the RTgutGC cell grown in a Transwell system under double seeding conditions retains comparable morphological characteristics (microvilli formation, metabolic activity in the form of expression of xenobiotic associated genes and similar metabolism of common metals) to the native tissue as demonstrated by Nadella et al. (2006a) without any modification. Although single seeding of Transwell inserts has previously been carried out using the RTgutGC cell line (Geppert et al. 2016) and is common among the culture of Caco-2 Transwell models, TEER is directly comparable between the single and double seeded approach despite differing culture times.

Our study is suggestive of an improved model under double seeding conditions comparable to “gut sac” methodology. This improved model would more readily support high throughout toxicity testing in future research. Indeed, the similarity of uptake kinetics between “gut sac” preparations and in vitro Transwell models (double seeded) is suggestive of a conservation of the Cu uptake pathway in the cell line. This conservation could allow for an increased understanding of dietary uptake and metabolism of environmentally relevant metals in addition to other contaminants. Indeed, unlike other aquatic models which require higher concentrations of toxicants to detect a toxic response (e.g. Schirmer, 2006), the RTgutGC model has shown itself to be akin to the standard gut sac technique predominantly used in the dietary toxicity studies (e.g. Nadella et al. 2006a, b). Further, to the establishment of comparable morphological developments via TEM, the RTgutGC system is also able to tolerate varying pH apical saline solutions which simulates in vivo scenarios, a finding previously observed for ex vivo cultures of gill epithelial (Stott et al. 2015). Mimicking the absorptive barriers found in native intestine, the RTgutGC cell line grown on Transwell inserts and modified as outlined in this study provides an avenue for examining the permeability of environmental toxicants in two large sections of the intestine as a replacement or supplement to in vivo animal tests in line with the tenet of the 3Rs. Currently, compounds with a high lipophilicity (log Kow < 4) have to be assessed under regulatory requirements for potential to bioaccumulate in aquatic systems using standard water or dietary routes (Lillicrap et al. 2016; OECD 2012). However, empirical experience would suggest that compounds between log Kow 3 and 4.5 would be of lower risk of accumulation and these could potentially be screened using a Transwell intestinal model such as that illustrated here, rather than using live fish. Our study goes some way towards achieving these goals in line with regulatory commitments and to support the 3Rs initiatives.

## Electronic supplementary material

Supplementary Information

Supplementary Information
