# Supplementary material for: Application of the rainbow trout derived intestinal cell line (RTgutGC) for ecotoxicological studies: molecular and cellular responses following exposure to copper
Source: Ecotoxicology. 2017 Aug 7;26(8):1117–33. doi: 10.1007/s10646-017-1838-8 (PMC5617881; doi:10.1007/s10646-017-1838-8)
Supplement: Supplementary file 1 — Supplementary Information [file 10646_2017_1838_MOESM1_ESM.docx]

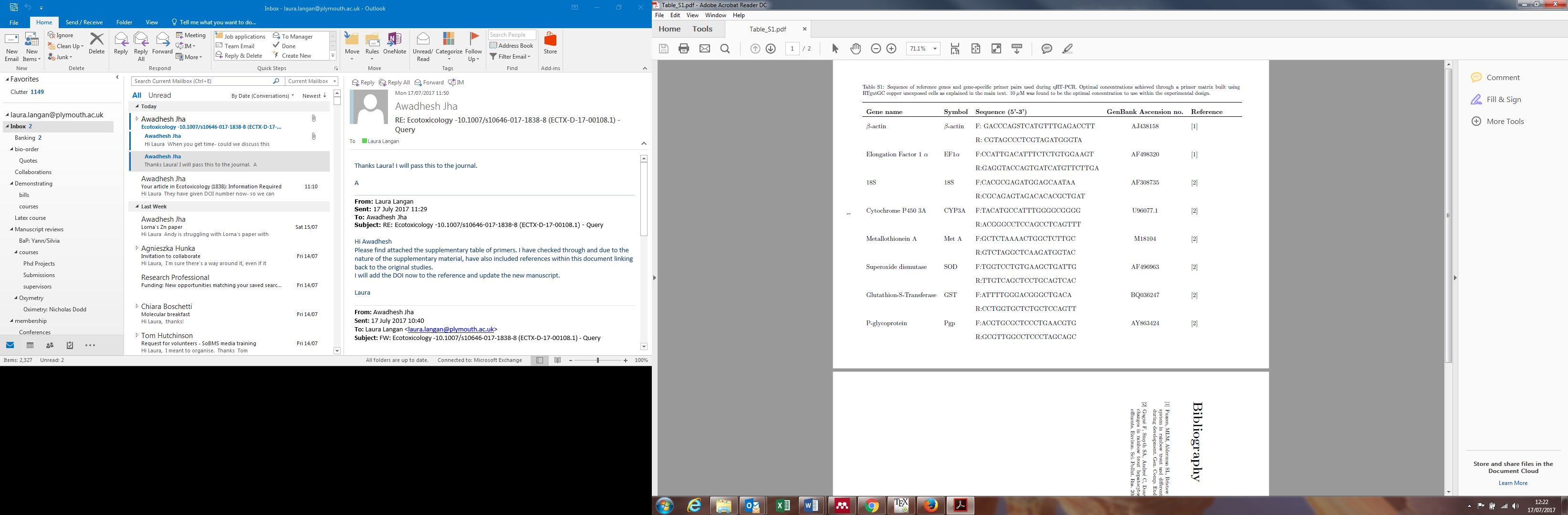


Table S1: Sequence of reference genes and gene-specific primer pairs used during qRT-PCR. Optimal concentrations achieved through a primer matrix built using RTgutGC copper unexposed cells as explained in the main text. 10 µM was found to be the optimal concentration to use within the experimental design.

**References**

1. Fuzzen, MLM, Alderman SL, Bristow EN, Bernier NJ (2011) Ontogeny of the corticotropin-releasing factor system in rainbow trout and differential effects of hypoxia on the endocrine and cellular stress responses during development. Gen. Comp. Endocrinol. 170: 604–612. doi: 10.1016/j.ygcen.2010.11.022
2. Gagné F, Smyth SA, Andre´e C, Douville M, Gélinas M, Barclay K (2013) Stress-related gene expression changes in rainbow trout hepatocytes exposed to various municipal wastewater treatment influents and effluents. Environ. Sci. Pollut. Res. 20: 1706–1718. doi: 10.1007/s11356-012-1174-8
