# Supplementary material for: Application of the rainbow trout derived intestinal cell line (RTgutGC) for ecotoxicological studies: molecular and cellular responses following exposure to copper
Source: Ecotoxicology. 2017 Aug 7;26(8):1117–33. doi: 10.1007/s10646-017-1838-8 (PMC5617881; doi:10.1007/s10646-017-1838-8)
Supplement: Supplementary file 2 — Supplementary Information [file 10646_2017_1838_MOESM2_ESM.pdf]

Table S1: Sequence of reference genes and gene-specific primer pairs used during qRT-PCR. Optimal concentrations achieved through a primer matrix built using RTgutGC copper unexposed cells as explained in the main text. 10 M was found to be the optimal concentration to use within the experimental design.

**Gene name**	**Symbol**	**Sequence (5'-3')**	**GenBank Ascension no.**	**Reference**	Con (μM)
β-actin	β-actin	F: GACCCAGSTCATGTTTGAGACCTT	AJ438158	Fuzzen et al., 2011	5
		R: CGTAGCCCTCGTAGATGGGTA			
Elongation Factor 1 α	EF1α	F:CCATTGACATTTCTCTGTGGAAGT	AF498320	Fuzzen et al., 2011	10
		R:GAGGTACCAGTGATCATGTTCTTGA			
18S	18S	F:CACGCGAGATGGAGCAATAA	AF308735	Gagné et al., 2013	10
		R:CGCAGAGTAGACACACGCTGAT			
Cytochrome P450 3A	CYP3A	F:TACATGCCATTTGGGGCGGGG	U96077.1	Gagné et al., 2013	10
		R:ACGGGCCTCCAGCCTCAGTTT			
Metallothionein A	Met A	F:GCTCTAAAACTGGCTCTTGC	M18104	Gagné et al., 2013	10
		R:GTCTAGGCTCAAGATGGTAC			
Superoxide dismutase	SOD	F:TGGTCCTGTGAAGCTGATTG	AF496963	Gagné et al., 2013	5
		R:TTGTCAGCTCCTGCAGTCAC			
Glutathion-S-Transferase	GST	F:ATTTTGGGACGGGCTGACA	BQ036247	Gagné et al., 2013	10
		R:CCTGGTGCTCTGCTCCAGTT			
P-glycoprotein	Pgp	F:ACGTGCGCTCCCTGAACGTG	AY863424	Gagné et al., 2013	10
		R:GCGTTGGCCTCCCTAGCAGC			
